# Supplementary figures and images for: Spatial assessment of advanced-stage diagnosis and lung cancer mortality in Brazil
Source: PLoS One. 2022 Mar 18;17(3):e0265321. doi: 10.1371/journal.pone.0265321 (PMC8932618; doi:10.1371/journal.pone.0265321)

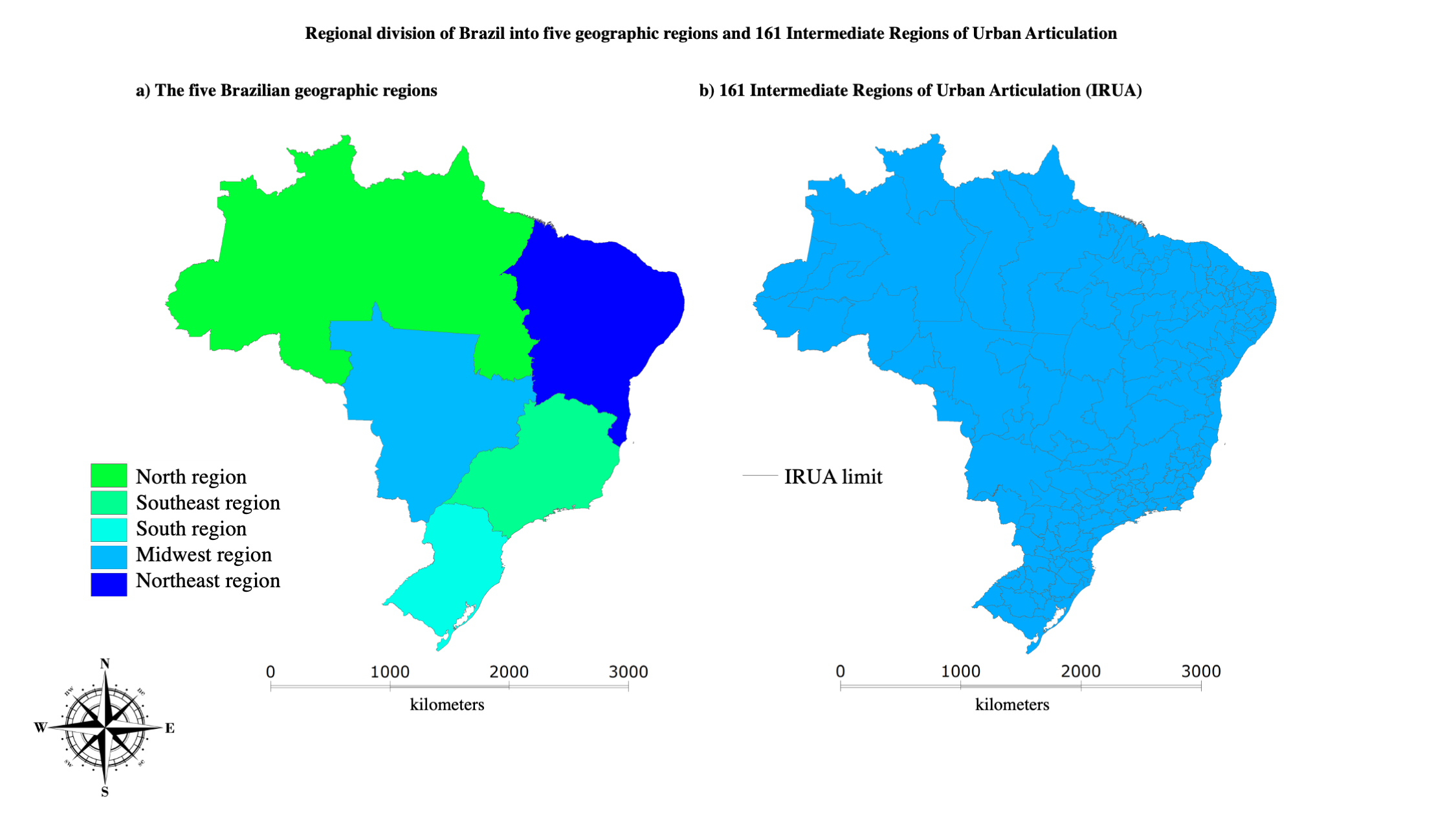

Supplement: S1 Fig — (TIF) [file pone.0265321.s001.tif]

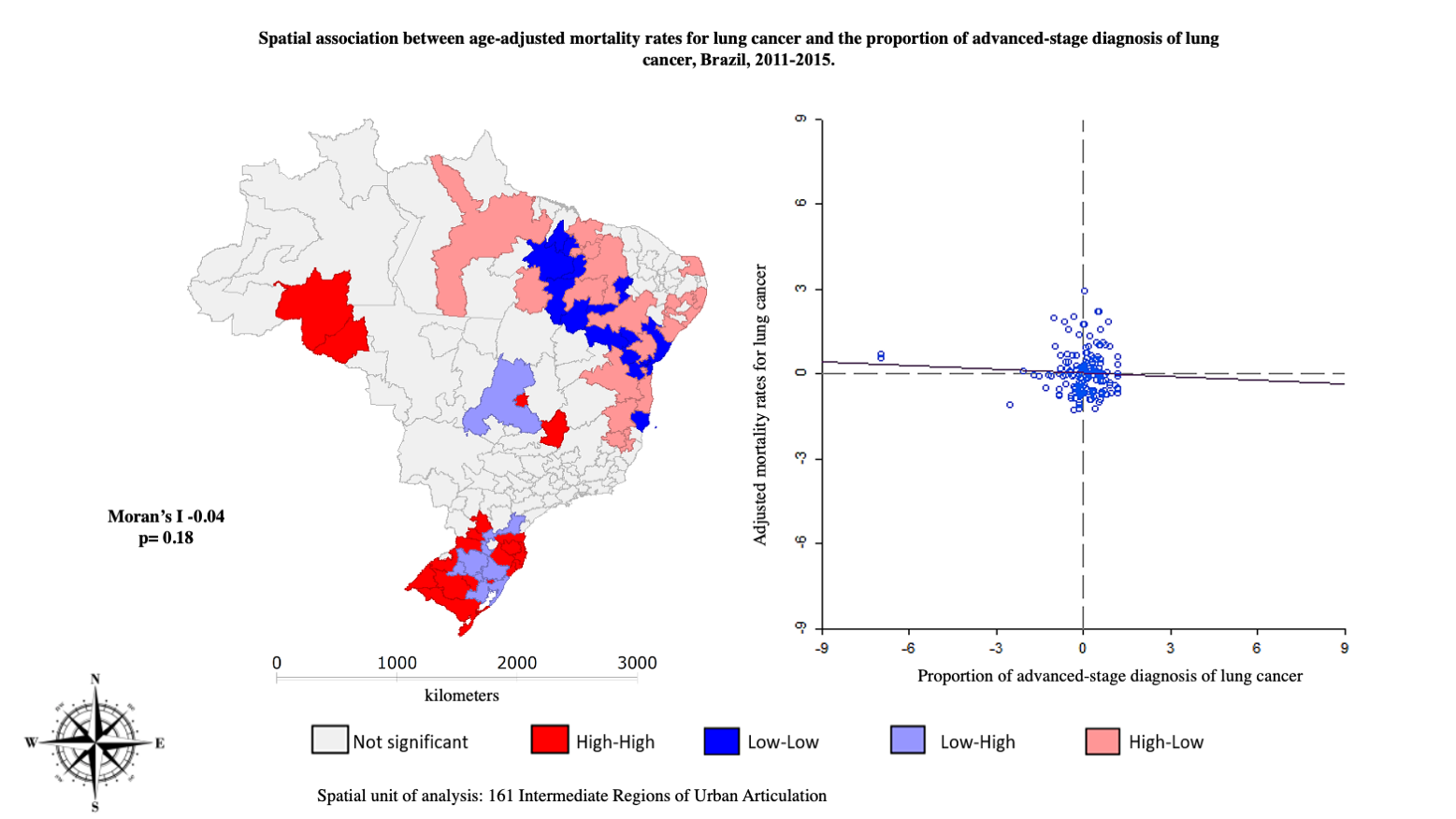

Supplement: S2 Fig — (TIF) [file pone.0265321.s002.tif]

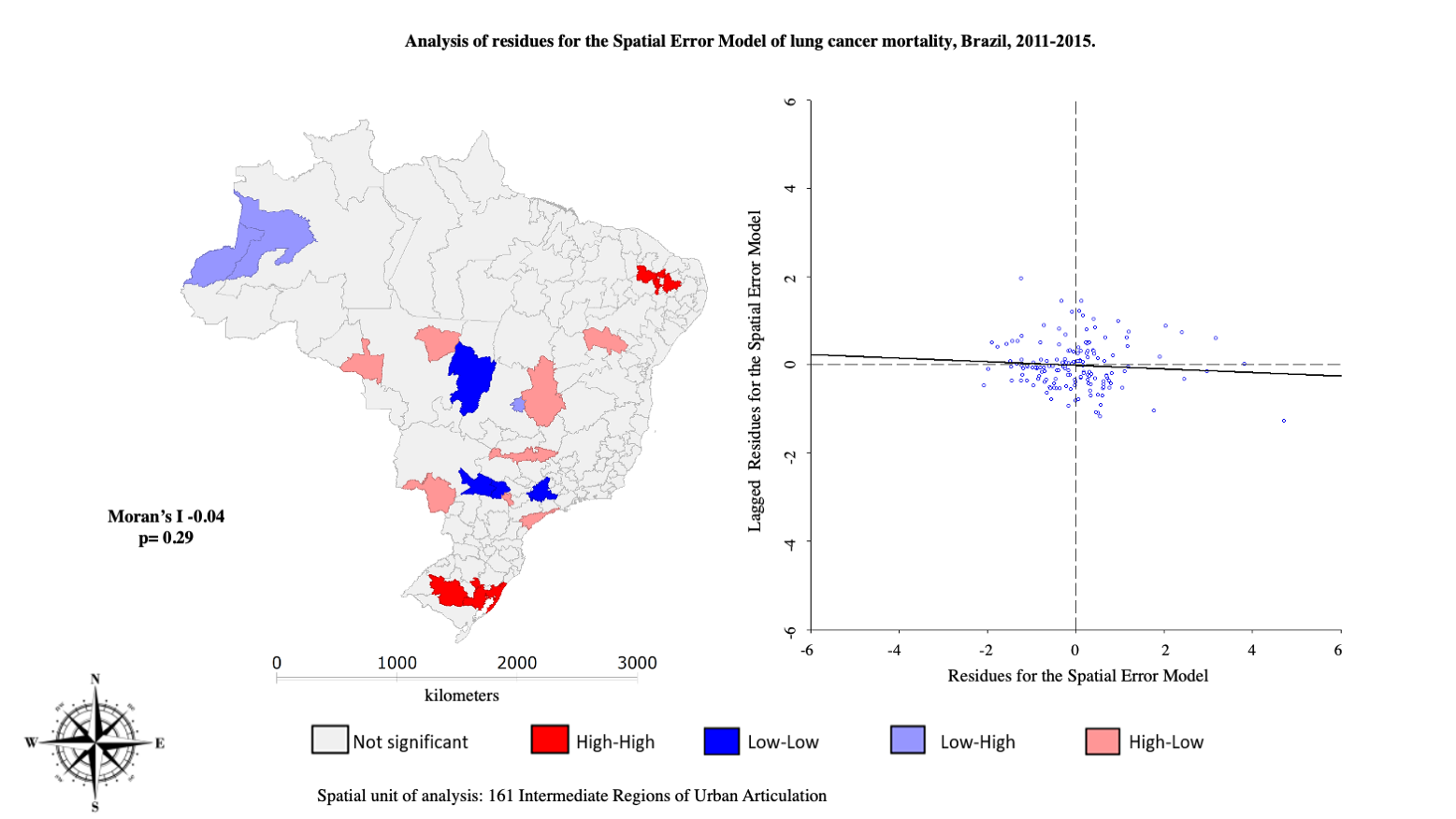

Supplement: S3 Fig — (TIF) [file pone.0265321.s003.tif]
