# Supplementary material for: Spatial assessment of advanced-stage diagnosis and lung cancer mortality in Brazil
Source: PLoS One. 2022 Mar 18;17(3):e0265321. doi: 10.1371/journal.pone.0265321 (PMC8932618; doi:10.1371/journal.pone.0265321)
Supplement: S1 Table — (DOCX) [file pone.0265321.s004.docx]

**S1 Table . Models of spatial regression, according to the analysis criteria for selecting the final model.**

| **Model** | **Akaike info criterion** | **Log likelihood** | **Schwarz criterion** | **R-squared** | **Lambda** |
| --- | --- | --- | --- | --- | --- |
| Spatial error | 824.113 | -408.056 | 836.438 | 0.663 | 0.787 |
| Classic | 860.531 | -426.265 | 872.856 | 0.552 | - |
| Spatial lag | 841.862 | -415.931 | 857.269 | 0.612 | 0.468 |
