# Supplementary material for: Spatial assessment of advanced-stage diagnosis and lung cancer mortality in Brazil
Source: PLoS One. 2022 Mar 18;17(3):e0265321. doi: 10.1371/journal.pone.0265321 (PMC8932618; doi:10.1371/journal.pone.0265321)
Supplement: Attachment — Submitted filename: REVIEW_Manuscript Number PONE-D-21-20112.pdf [file pone.0265321.s005.pdf]

REVIEW – PLOS ONE

DATE: 08/29/2021 - Aug 29 2021

TITLE OF THE MANUSCRIPT: Spatial assessment of advanced stage diagnosis and lung cancer mortality in Brazil

REVIEWER: Alessandra Cristina Guedes Pellini

The article deals with a relevant problem for Public Health, in terms of magnitude and mortality – lung cancer. It uses spatial analysis techniques to explore ecological-level relationships between advanced-stage diagnosis and lung cancer mortality (treated as dependent variables in this study) and some socioeconomic indicators and others indicators related to the provision of health services. The units of analysis chosen were the Intermediate Regions of Urban Articulation in Brazil (IRUA). The authors use global and local spatial analysis methodologies as well as multivariate analysis to examine relationships of interest.

The study is relevant, interesting and treats the literature in an adequate and coherent way. However, some points can be improved, and this review has as its main foundation a perspective of work improvement.

SOME GENERAL ISSUES:

1. Dependent and Independent Variables:

- ✓ The authors bring together some independent variables of the study into a group called "socioeconomic indicators". However, the indicators that compose it are, in part, demographic (for example: index of aging and degree of urbanization) and, in part, socioeconomic, for example: Gini Index, Income). Thus, I suggest using the term "demographic and socioeconomic indicators", instead of just "socioeconomic indicators", changing every time they appear in the text. For any questions, consult:

  https://seriesestatisticas.ibge.gov.br/lista_tema.aspx?de=33&no=10&op=0
  https://seriesestatisticas.ibge.gov.br/lista_tema.aspx?op=0&de=94&no=16
  https://biblioteca.ibge.gov.br/visualizacao/livros/liv101678.pdf

2. Standardization of terms:

- ✓ It is worth clarifying that the term "correlation" is being referred to in the text as an autocorrelation or spatial dependence.
- ✓ Standardize the use of the term – "neoplasm" or "cancer" – throughout the text. The neoplasm can be malignant or benign, and it is called "câncer" when it is malignant. I therefore suggest always using the word "cancer".

3. Figures:

- ✓ Some maps lack essential cartographic elements, such as North arrow (Figures 1, S1 and S2), scale bar (all Figures – 1 to 5, S1 and S2); description of the spatial unit of analysis in the legend (all Figures).

- ✓ The standardization of colors in Fig. 1, in Moran Lisa Local maps, is different from the others. As it is a qualitative variable, I suggest following the same pattern for all of them, with the color pattern in Figures 2 to 5 discriminating better.
- ✓ Also take care of the shade of colors, for example, in Figures 3, 4 and 5 the shade of red (High-High category) of the legends is different from what is observed on maps.
- ✓ In the graphics that accompany the maps, I suggest increasing the font size of the X-axis labels and Moran's I values above the graphics. Also pay attention to the correct rounding of the Moran values presented below the maps, with only two decimal places, which must be in accordance with the values presented above the graphs.
- ✓ It is always interesting to describe on the map what each one is, it can be in the upper right corner – for example, Gini index, Aging rate, Income, etc.
- ✓ Specifically in Fig. 3, the Y axis of the last graph has a different title from the others.
- ✓ Also improve the axes titles of the residual analysis graph (Fig. S2).
- ✓ In Fig. 1, review the choice of criteria for the categories in the legend in Fig. 1a, as it has little discrimination. The first category ranges from 0 to 81, and the others range from 81 to 100. That is, in the first group there are units with proportions from 0 to 80... which does not help to discriminate the areas on the map, and hinders the discussion. I suggest redoing this categorization. For example, in the far west of the North region, the impression it gives is that it is spared, but in fact it is because it covers values between 0 and 80. Then it becomes more difficult to discuss, especially when, in the Discussion (Paragraph #5), the authors say that "In this study, it was observed that the diagnosis of lung cancer at an advanced stage is prevalent in almost the entire Brazilian territory".
- ✓ The criteria for choosing the categories of quantitative maps (Proportion and Mortality) must be included in the Methods Section – eg. quantiles, equal intervals, natural breaks, etc.
- ✓ In all maps, include the layer of the Regions of Brazil, in order to help in the discussion of the findings.
- ✓ In the titles of all Figures, after "per IRUA", add "Brazil, 2011-2015."
- ✓ In the titles of Fig. 1, where it refers to "combined per sex", substitute "for both sexes".

SOME SPECIFIC ISSUES:

1. Summary:

- ✓ Describe the "Objective" exactly as it appears in the body of the text, at the end of the Introduction Section.
- ✓ When presenting the results of the multiple analysis, describe how the indicators were related to the outcome (standardized mortality), and whether it was positive or negative and significant or not.
- ✓ Also describe the results of autocorrelation for Mortality (which socioeconomic and demographic, and health care offer variables were significantly correlated), as well as was this was done for the outcome of Proportion of diagnosis in advanced stage.

2. Introduction:

- ✓ Paragraph #1: the authors describe that "lung cancer is the second most incident type of cancer worldwide". - What is the first type of cancer? Include in text.
- ✓ Paragraph #4: the authors refer, at the end, that "The importance of each factor, however, varies with the different disease scenarios for lung cancer". - Make it clearer what these different disease scenarios are.

3. Methods:

- ✓ Paragraph #1: justify the choice of "Intermediate Regions of Urban Articulation (IRUA)", as the spatial analysis unit.
- ✓ Paragraph #3: the authors state that: "The IRUA are a type of Urban-Regional Division, which organize the territory into metropoles, regional capitals, and smaller urban centers, considering the flows of public and private management and the existence of more complex urban functions, without necessarily following the limits of FU". - In relation specifically to "public and private management flows", what kind of sectors do the authors refer to? Health only or others? Clarify.
- ✓ Paragraph #3: specify here, too, whether the IRUA boundaries coincide with the UF and municipal boundaries. Given that data (dependent and independent variables) were obtained from municipalities, explain how these data were converted or transformed for the IRUA spatial analysis units. (This is commented a little at the end of the seventh paragraph of Methods, but it would be interesting to bring it here and complement). Do these units of analysis make up a digital territorial mesh available at IBGE? Clarify in the text.
- ✓ Paragraph #4: "The dependent variables were the proportion of advanced-stage diagnosis and the adjusted mortality rates for lung cancer, combined for men and women, per IRUA, for 2011-2015." - specify how the adjustment of the mortality rate was made. By gender and age group?
- ✓ Paragraphs #6 and #7: these paragraphs discuss the information sources of the dependent variables; therefore, they should come before the fifth paragraph (which deals with independent variables)
- ✓ Paragraph #7: presents the ICD-10 C33-C34, however, the ICD-10 C33 is from tracheal cancer. It is important to clarify that whenever the text mentions lung cancer, it is also including tracheal cancer (ICD-10: C33), in addition to bronchial and lung cancers (ICD-10: C34 to C34.9).
- ✓ Paragraph #8: describe in full what the acronym UNDP means. It also needs to better specify the source of each of the socioeconomic/demographic variables and the variables related to health care. Specify for each variable. If you prefer, put it in a table format, containing each variable, its source, the indicator calculation method and the variable name used in the maps and in the multiple analysis. It was also unclear why the population by sex and age was collected for these variables, since there was no separate analysis of these indicators by sex or age.
- ✓ Paragraph #8: the authors state that "The calculation of indicators employed as the denominator the population of the 2010 census, per municipality, sex, and age as reported by IBGE." Why was not used a more recent population that would correspond to the middle of the evaluated period – 2011 to 2015? For example, on the DATASUS website, it is possible

to obtain the population estimate for 2012, by age group and municipality – this year would be closer in relation to the other events in the study. See: http://tabnet.datasus.gov.br/cgi/deftohtm.exe?ibge/cnv/popbr.def

- ✓ Paragraph #9: "Cases of carcinoma In Situ (TNM 0) and with no staging indication and residence were excluded." - It is important for the authors to discriminate, in the Results, how many cases (N and %) represented carcinoma in situ, how many cases had no staging indication, and how many cases did not contain place of residence. In general, in cancer registries, we have difficulty calculating incidence for smaller locations, due to gaps in case address data. I believe it is not the case with information about the municipality of residence, but it is worth sizing up. This paragraph would look better with those referring to dependent variables. In the Results, it would be important to inform how much the proportions of advanced-stage diagnosis of lung cancer varied between the IRUA (minimum and maximum values and where they occur), as well as how much the mortality rates varied (minimum and maximum and where they occur).
- ✓ Paragraph #10: "The 2013 population was employed as a reference and collected from the population estimate: municipality, sex, and age, as reported by IBGE." - This was confused by the previous statement (in the Paragraph # 8) that "The calculation of indicators employed as the denominator the population of the 2010 census, per municipality, sex, and age as reported by IBGE." - If this last information refers to the calculation of socioeconomic and demographic indicators, it needs to clarify the reason for this option.
- ✓ Paragraph #10: The crude and adjusted mortality rates (AMR) (per 100,000 inhabitants) were calculated for each IRUA according to the world population". - Specify here how the adjustment was made (by sex and age group?), and rewrite so that it is clear that the world standard population was used only for the calculation of the adjusted rates (and not for the crud rates).
- ✓ Paragraph #11: "Global Moran's Index and the Local Indicator of Spatial Association (LISA) were employed to verify spatial correlation and its significant patterns, respectively." - In fact, the Moran Global Index assesses whether there is spatial dependence on the space as a whole, and the Moran LISA assesses these relationships locally, in some locations showing significance and not in others. So, rewrite this sentence, which is confusing (I suggest reviewing the concepts and uses of Moran Global and Local at: http://www.panaftosa.org.br/Comp/MAPA/442913.pdf)
- ✓ Paragraph #11: It is important to clarify that the authors use the Moran Local Univariate for each dependent variable, and analyze the Moran Local Bivariate between the independent and dependent variables. This needs to be clarified, as well as in the low-high and high-low patterns, which order is specified – VD-VI or VI-VD. Also specify what the graphics that appear beside each map mean.
- ✓ Paragraph #12: why was multiple analysis done only for mortality, but not for the proportion of lung cancer cases with advanced diagnosis?
- ✓ Paragraph #12: the last sentence "The final multivariate model included statistically significant variables and those with theoretical plausibility." - is in disagreement with the first: "Multivariate analysis of lung cancer mortality included only the independent variables that presented a statistically significant correlation with the dependent variable". It seems to me that only the last one in the paragraph is correct, as the final model kept only one variable with theoretical plausibility. It is also important to say with which models the spatial error model was compared, and to refer to the S1 table.

4. Results:

- ✓ Paragraph #1: include information on maximum and minimum variations (and respective locations where they were found) of proportions (PASD) and mortality. When it is said that "The formation of only one low-low cluster located in the North region was observed.", it would also be important to refer to the other high-high clusters found.
- ✓ Paragraph #2: specify how many more cases the correction was able to add to the total N of deaths, that is, why is this correction work important?
- ✓ Paragraph #2: where it reads "For the combined AMR for men and women, the average was 12.82 (SD 5.12) deaths per 100,000 inhabitants.", replace with**: "The AMR including men and women** was 12.82 (SD 5.12) deaths per 100,000 inhabitants". As it stands, it looks like an average of the rates for men and women was taken... would that be the case?
- ✓ Paragraph #3: "The independent socioeconomic variables demonstrated low spatial correlation with advanced -stage diagnosis, with statistical significance for Gini (p=0.01), aging rate (p=0.02), and income (p=0.01)" - also include the values of the Moran's I. About the statement: "Regarding the variables related to the offer and availability of health services, no statistically significant correlations were observed with proportion of advanced-stage diagnosis, as displayed in Fig 3". - specify that no global spatial dependence was found (as at local levels they were observed, as seen in LISA maps).
- ✓ Paragraph #4: "Fig 4 and 5 show the correlation between AMR, combined by sex, for lung cancer and the socioeconomic and health service offer indicators. - Replace with: "Fig 4 and 5 show the correlation between AMR, for both sex, for lung cancer and the socioeconomic and health service offer indicators, **respectively."**
- ✓ Table 1: Consider revising the title to: **"Spatial regression analysis of adjusted lung cancer mortality rates and its correlation whit socioeconomic and health service offer indicators, per IRUA, Brazil, 2011-2015."** At the bottom of the table, there appears to be an error, as what is itemized as "p = 0.663" looks like it would have to be "R^2 = 0.663".
- ✓ Paragraph #6: Replace "was preserved" by "was kept in the model.".

5. Discussion:

- ✓ Paragraph #3: "Although there is consensus in the literature on the histological types of lung cancer with a higher risk of diagnosis at an advanced stage, there are still inconsistent results regarding the effect of socioeconomic conditions and the provision of health services on the stage of the neoplasia in the diagnosis" – Better explain what the authors mean by **"there are still inconsistent results",** that is, what is the knowledge gap in relation to what is stated, or discuss in accordance with other references.
- ✓ Paragraph #5: Replace "PASD were positively correlated with the aging rate and income and inversely correlated with Gini" with "PASD were positively correlated with the aging rate and income and inversely correlated with **Gini Index"**.
- ✓ Paragraph #7: "...encompassing the North and Northeast regions, with the worst socioeconomic índices" – Highlight the findings in Fig. 2 (low-low pattern for the North Region) and Fig. 4 (low-low pattern for the North and Northeast region), with regard to income and urbanization.
- ✓ Paragraph #8: It is better not to talk about **incidence,** as this indicator was not evaluated in the study. As for considerations about reducing the smoking habit in Brazil, and its impact

on the incidence of lung cancer, it is worth discussing in light of the findings of the Vigitel studies and INCA: https://www.inca.gov.br/observatorio-da-politica-nacional-de-controle-do-tabaco/dados-e-numeros-prevalencia-tabagismo

- ✓ Paragraph #8: At the end of the paragraph, it is stated that: "...the proportion of cases at advanced stages can accompany the incidence of lung cancer at the Brazilian IRUAs." I believe this statement is fragile, given, above all, that the diagnostic capacity and resources are different in different regions of Brazil.
- ✓ Paragraph #13: Likewise, one should avoid talking about **"incidence",** but report the evaluated indicator – "proportions of advanced-stage diagnosis of lung câncer".
- ✓ Paragraph #15: "Regarding the offer and availability of health services, in this study, the high mortality rates were correlated with territories that presented low coverage of health plans and high density of facilities licensed in oncology." - This sentence expresses, in part, the findings of the spatial regression analysis, referring to the "health plan coverage"; but not in relation to the "high density of facilities licensed in oncology", which was not significant, and was maintained only for conceptual reasons. If analyzed from the point of view of Fig. 5, spatial dependence was positive in the bivariate analysis, in relation to the coverage of health plans, as well as in relation to the high density of oncology services. A possible explanation may be due to the fact that these areas are sought after by people seeking treatment for their cancer, who may even take up residence in them. It is important to discuss these points more clearly, and about the discrepancy between the findings in the different analyzes (Spatial regression analysis and Moran's I).
- ✓ Paragraph #18: "In the South, the research evidenced IRUA with high mortality rates for lung cancer and low coverage of health plans." - This is an assertion resulting from the finding expressed in Fig. 5, whose legend indicates that the South region has a low-high standard in relation to the health plan coverage; hence the need to clarify, in the Methods Section, that the first indicator – low – refers to the independent variable (in this case – health plan coverage) and the second – high – refers to mortality rates; that is, make it clearer, so as not to give the opposite impression. It can be clarified in the Methods (that the first word of the pattern refers to VI and the second to VD).
- ✓ Paragraph #20: "...and areas with a more comprehensive offer and availability of health facilities licensed in oncology could result from high number of diagnoses of the disease, more structured surveillance and better quality death records in these places"– adding to this the fact that people can take up residence in these locations in order to facilitate their access to treatment.
- ✓ Paragraph #21: "Eight presented higher standards, five were compatible, and 16 presented lower standards than suggested by the Therapeutic Directives." - How many of these 16 services were exclusively of Supplementary Health? Considering that many private services also provide services to the SUS...
- ✓ Paragraph #22: "These differences emphasize that most treatment centers offer lower-quality care than preconized by the Ministry of Health and provided by Supplementary Health." – It is not clear whether SUS establishments follow the Ministry of Health Therapeutic Directives less and that Supplementary Health performs better in this regard. Apparently this is what is said, but it must be justified, if possible, on the basis of numbers. In the previous paragraph, this is also not very clear. Please rewiew.
- ✓ Paragraph #24: "The analysis shows that the spatial distribution of the proportion of advanced stage lung cancer diagnosis in the Brazilian IRUA is not significantly affected by

contextual socioeconomic factors and the offer of health services". – This statement is in disagreement with Fig. 2, which shows a significant bivariate correlation between the indicator "proportion of advanced stage lung cancer diagnosis" and most of the analyzed socioeconomic and demographic factors, except the "Urbanization degree".

- ✓ Paragraph #25: "The cases excluded, due to the absence of data, could present a non-random spatial distribution, which was not assessed herein" – again, it is important to include how many of these cases were excluded due to lack of data, that is, how much of the sample, in %, they represented.

6. Conclusion:

- ✓ Paragraph #1: "... The PASD were weakly correlated to socioeconomic indicators only." – Consider replacing with: "The PASD were weakly **spatially** correlated to socioeconomic indicators only."
