# Supplementary material for: Spatial assessment of advanced-stage diagnosis and lung cancer mortality in Brazil
Source: PLoS One. 2022 Mar 18;17(3):e0265321. doi: 10.1371/journal.pone.0265321 (PMC8932618; doi:10.1371/journal.pone.0265321)
Supplement: Attachment — Submitted filename: Comments for Plos One PONE-D-21-20112 V2.docx [file pone.0265321.s007.docx]

**Comments for Plos One - Manuscript entitled 'Spatial assessment of advanced stage diagnosis and lung cancer mortality in Brazil' (PONE-D-21-20112-R1)**

In their manuscript, Lima et al. provided relevant and innovative information regarding the spatial distribution of advanced stage at diagnosis and mortality of lung cancer patients and its correlation with the supply of healthcare services and socioeconomic indicators in Brazil. I consider that the new version (R1) of the manuscript improved considerably and that the authors answered all points made by reviewers. I have only some small comments regarding the manuscript:

**Authors´ affiliation**

1. I think there is an error with the affiliation of the first author as in the manuscript appears two different Postgraduate Programs: 'Graduate Program in Collective Health' and 'Graduate Program in Public Health' belonging to the same Department (Public Health or Collective Health?).

**Abstract**

2. I would like to suggest rewriting the sentence 'The adjusted mortality rates for lung cancer presented positive and statistically significant correlation with all the demographic and socioeconomic and health service offer indicators [...].' to 'The adjusted mortality rates for lung cancer presented a positive and statistically significant correlation with all demographic, socioeconomic and healthcare services supply indicators [...].'

The term 'health service offer' appears many times in the manuscript, I would like to suggest considering using 'healthcare services supply' or 'supply of healthcare services'.

3. I would like to suggest to change the term 'Health plan coverage' to 'private health insurance coverage'. The authors sometimes also use the term 'Supplementary Health', I suggest unifying them.

**Introduction**

4. I would like to suggest to chance the term 'advanced stage diagnosis' to 'advanced-stage diagnosis' or to 'advanced stage at diagnosis'.

**Materials and methods**

5. Sometimes, the authors use the term 'Federation Units (FU)' other 'states'. Foreign people do not know if they are the same or not. Please, consider unifying them.

The same problem happens to 'Integrator of Hospital Cancer Records'. Sometimes the authors use 'Integrator' other 'RHC' or 'IRHC' or 'RHC Integrator'. I suggest using the abbreviation 'Integrator-HBCRs' to the term 'Integrator of Hospital-Based Cancer Registries'.

6. Lung cancer is morphologically classified into different subtypes. Because of this, I suggest changing the term 'carcinoma in situ' to 'in situ disease'.

7. Please correct the spelling of 'non-colinear variables' to 'non-collinear variables'.

**Results**

8. I would like to suggest changing the term 'lung cancer adjusted mortality rates' to 'age-adjusted mortality rates for lung cancer' or to 'age-adjusted lung cancer mortality rates'.

**Figure 1**

9. I would like to suggest changing the term 'without staging' to 'stage not known'.

10. I would like to suggest changing the term 'carcinoma in situ' to 'in situ disease' or to 'not invasive cancer'.

11. Please, change the sentence '31,010 (73.2%) cases of lung cancer (age between 18 and 99 years were included)' to '31,010 (73.2%) cases of lung cancer (age between 18 and 99 years) were included'.

**S2 Fig**

12. Include in the title the term 'Brazil'.

**S3 Fig**

13. Include in the title the term 'Brazil'. Further, include the information about the meaning of x-axis and y-axis.

**Table 1**

14. Include in the footnote the meaning of the abbreviations used in the table

15. I would like the confirmation of the authors if the description they used for the variable 'adjusted mortality rates for lung cancer' is correct: 'Lung cancer mortality rate, combined for men and women, adjusted for age and standard world population'.
