# Supplementary material for: Spatial assessment of advanced-stage diagnosis and lung cancer mortality in Brazil
Source: PLoS One. 2022 Mar 18;17(3):e0265321. doi: 10.1371/journal.pone.0265321 (PMC8932618; doi:10.1371/journal.pone.0265321)
Supplement: Attachment — Submitted filename: Response to reviewers.docx [file pone.0265321.s008.docx]

**Response to reviewers**

Additional Editor Comments:

I commend the authors for the significant improvement related to the new version of their manuscript. However, there are still several issues that must be addressed before it can be reconsidered for publication, as described by the reviewers.

In their first report on this manuscript, reviewer #3 argued that the authors had often misused the term “correlation” in their text. The reviewer claimed that correlation measures do not suggest any direction regarding the relationship between two variables. On the one hand, that explanation is incorrect because correlation coefficients measure the extent to which two variables have a linear relationship and may be positive or negative, thereby indicating the direction of that relationship. On the other hand, the authors responded to the reviewer’s comment by arguing that “The term association is used with qualitative variables. When variables are quantitative, as in the case of this study, the term correlation is the most appropriate. When testing the correlation, the coefficient generated quantifies the strength and direction of the relationship between two numerical variables, with results ranging from −1 to 1, which indicate a positive or negative correlation. Thus, when considering the type of variables included in the study, the term correlation is the most recommended.” Unfortunately, the authors’ argument also has several important flaws. First, it is incorrect to claim that the word “association” should be restricted to the relationship between qualitative variables. Although association and correlation are sometimes loosely used as synonyms, in statistics, correlation is more commonly used to refer to correlation coefficients, whereas association is a more generic term denoting a statistical dependence between two or more variables that may also be positive or negative. Moreover, correlation coefficients reflect the degree of linear association, i.e. the extent to which the points of the scatter plot of two variables are close to a monotonic line but say nothing about the slope of that line, which would be better described the coefficient of a linear regression equation. Hence, for the sake of clarity, I would like to recommend that the authors restrict the use of the term correlation to situations where they refer specifically to correlation coefficients or spatial autocorrelation measures and that they use the term association when referring to the coefficients derived from the equations of bivariate and “multivariate” spatial regression analyses throughout the text. By the way, “multivariable” regression is a more accurate term for the analyses performed in this study than “multivariate” regression. See the following article by Hidalgo and Goodman (https://www.ncbi.nlm.nih.gov/pmc/articles/PMC3518362/)

With regards to the association vs. correlation nomenclature, I recommend checking Miguel Porta’s Dictionary of Epidemiology (https://www.oxfordreference.com/view/10.1093/acref/9780199976720.001.0001/acref-9780199976720). As to the interpretation of correlation coefficients, I would like to suggest the following article by Patrick Schober (<https://pubmed.ncbi.nlm.nih.gov/29481436/>)

We acknowledge the reviewer’s comments and the terms were corrected.

Reviewer #1:

All my comments have been addressed and I consider that the manuscript is suitable for publication in this version.

Reviewer #2:

Authors´ affiliation

1. I think there is an error with the affiliation of the first author as in the manuscript appears two different Postgraduate Programs: 'Graduate Program in Collective Health' and 'Graduate Program in Public Health' belonging to the same Department (Public Health or Collective Health?).

The correct affiliation is Department of Collective Health, Postgraduate Programme in Collective Health. It has been corrected.

Abstract
2. I would like to suggest rewriting the sentence 'The adjusted mortality rates for lung cancer presented positive and statistically significant correlation with all the demographic and socioeconomic and health service offer indicators [...].' to 'The adjusted mortality rates for lung cancer presented a positive and statistically significant correlation with all demographic, socioeconomic and healthcare services supply indicators [...].'
The term 'health service offer' appears many times in the manuscript, I would like to suggest considering using 'healthcare services supply' or 'supply of healthcare services'.

We acknowledge the reviewer’s comment and the “health service offer” was replaced by “healthcare services supply” in the manuscript.

3. I would like to suggest to change the term 'Health plan coverage' to 'private health insurance coverage'. The authors sometimes also use the term 'Supplementary Health', I suggest unifying them.

We appreciate the suggestion. The authors chose to unify the term to 'Health plan coverage'.
The description about the origin and calculation of the variable are in the table of variables.

Introduction
4. I would like to suggest to chance the term 'advanced stage diagnosis' to 'advanced-stage diagnosis' or to 'advanced stage at diagnosis'.

The term has been changed to 'advanced-stage diagnosis'.

Materials and methods

5. Sometimes, the authors use the term 'Federation Units (FU)' other 'states'. Foreign people do not know if they are the same or not. Please, consider unifying them.
The same problem happens to 'Integrator of Hospital Cancer Records'. Sometimes the authors use 'Integrator' other 'RHC' or 'IRHC' or 'RHC Integrator'. I suggest using the abbreviation 'Integrator-HBCRs' to the term 'Integrator of Hospital-Based Cancer Registries'.

The term been unified to 'Federation Units (FU)' and the abbreviation “ Integrator of Hospital-Based Cancer Registries (Integrator-HBCRs)” was employed.

6. Lung cancer is morphologically classified into different subtypes. Because of this, I suggest changing the term 'carcinoma in situ' to 'in situ disease'.

The text has been changed as required.

7. Please correct the spelling of 'non-colinear variables' to 'non-collinear variables'.

The text has been changed to:

The multivariable analysis of lung cancer mortality included explanatory variables that showed a statistically significant correlation with the response variable and that were non-collinear with each other (correlation < 0.7).

Results
8. I would like to suggest changing the term 'lung cancer adjusted mortality rates' to 'age-adjusted mortality rates for lung cancer' or to 'age-adjusted lung cancer mortality rates'.

The term has been changed to 'age-adjusted mortality rates for lung cancer’.

Figure 1

9. I would like to suggest changing the term 'without staging' to 'stage not known'.

The term has been modified as requested.

10. I would like to suggest changing the term 'carcinoma in situ' to 'in situ disease' or to 'not invasive cancer'.

The term has been changed in the text and figure.

11. Please, change the sentence '31,010 (73.2%) cases of lung cancer (age between 18 and 99 years were included)' to '31,010 (73.2%) cases of lung cancer (age between 18 and 99 years) were included'.

The sentence has been modified as required.

S2 Fig

12. Include in the title the term 'Brazil'.

The title has been included.

S3 Fig

13. Include in the title the term 'Brazil'. Further, include the information about the meaning of

x-axis and y-axis.

Axis names have been changed to full form (Residues for the Spatial Error Model and Lagged Residues for the Spatial Error Model).

Table 1

14. Include in the footnote the meaning of the abbreviations used in the table.

The footnote has been included below the table.

15. I would like the confirmation of the authors if the description they used for the variable 'adjusted mortality rates for lung cancer' is correct: 'Lung cancer mortality rate, combined for men and women, adjusted for age and standard world population'.

The description has been modified as requested by reviewer 4.

Reviewer #3:

In relation to the topic 'correlation' and its use, I was not referring to the measure that varies between -1 and 1 and which is a correlation. The author appears to be using the term correlation when it is a measure of correlation and when it is not. For example, in the title of Figure 2, 'Spatial regression analysis of adjusted lung cancer mortality rates and their correlation with socioeconomic indicators and health service provision, by IRUA, 2011-2015', the author used the term correlation for the coefficients estimated by the regression model, this measure does not vary between -1 and 1, and it is not a correlation. It is more appropriate to use the term association between adjusted mortality and socioeconomic indicator rather than correlation.
Can the author please review?

The text has been reviewed and the terms has been corrected.

Reviewer #4:

The manuscript showed substantial improvement after the first review; however, I noticed that some aspects can still be improved. The new suggestions that I present, although they are relevant in number, are easy to resolve.

1. Figures:

⎫ Fig. 1: change the acronym “RHC”, which is in Portuguese, for “HCR” (Hospital Cancer Records).

The term has been changed to Integrator of Hospital-Based Cancer Registries (Integrator-HBCRs) as requested.

⎫ S1 table: fix the name of the “spatial lag” model (this is described as “spatial log”).

The name has been corrected.

2.Abstract:
⎫ Standardize the number of decimal places. For example: in lines 40 and 41 of the Abstract: “The proportion of advanced-stage diagnosis was 85.28% (95% CI 83.3-87.1).” – The proportion value (85.28%) appears with two places, and the 95% confidence interval with only one (95% CI 83.3-87.1). Standardize (I suggest keeping everything to two decimal places, then getting the CI values right). Also check this in the rest of the text.

The numbers has been standardized as requested.

⎫ Line 43: replace “Gini” by “Gini Index”.

The term has been changed.

⎫ Line 45: replace “significant correlation” by “significant spatial correlation”.

The term spatial has been inserted.

⎫ Lines 48 and 52 differ in the denomination of the variable related to Oncology Services – in line 48, it is described as “Density of licensed facilities in oncology”, and in line 52, “Density of Licensed Oncology Health Facilities”. Standardize the term, as well as the rest of the text.

The term has been standardized to density of facilities licensed in oncology.

⎫ Lines 51-53: replace the sentence: “The variable Density of Licensed Oncology Health Facilities presented positive correlation with the studied outcome, although without statistical significance.” by: “The variable Density of Licensed Oncology Health Facilities showed no significant correlation with lung cancer mortality rate”.

The sentence has been changed as required.

3. Introduction:

⎫ Line 62: the term “neoplasm” still appears in the text. Change the term “neoplasms” to “cancers” (including in reference [1] – GLOBOCAN, it is described as “cancers” and not “neoplasms”).

The term has been changed in the text.

⎫ Lines 64-66: “In Brazil, in 2019, there were 16,661 deaths due to lung cancer in men and 12,593 deaths in women, which correspond to an estimated risk of 16.19/100,000 men and 9,84/100,000 women [2]”. First the author presents the numbers of deaths, then he says that this corresponds to estimates of risks (incidences). Review if the data really correspond to the incidence, in this case, do not use the term “which correspond”, but treat the two sentences as independent. Another option would be to present the number of new cancer cases of each sex, and then correlate the respective risks.

The text has been changed to : In Brazil, in 2019, there were 16,661 deaths from lung cancer in men and 12,593 deaths in women, which correspond to an age-adjusted mortality rate of 16.19 per 100,000 men and 9.84 per 100,000 for women [2].

⎫ Lines 83-84: replace the phrase: “When considering the socioeconomic and distribution differences of health services in Brazilian regions” by: “When considering socioeconomic differences, as well as the distribution of health services in Brazilian regions”.

The phrase has been replaced as requested.

4. Material and Methods:

⎫ I suggest changing the name of the Subsection: “Study design” to “Study design and Spatial analysis units”, since the authors describe, in this item, several aspects related to the analysis units chosen for the study – IRUA.

The name has been changed.

⎫ In the subsection: “Study variables and data sources”, I suggest changing the order of the paragraphs, initially describing everything that refers to the response variables and, later, everything that concerns the exploratory variables. So, the new order would be:
♣ Paragraph #1: “The response variables were the proportion of late-stage diagnosis and adjusted mortality rates ...”
♣ Paragraph #2: “All data included here were collected by municipality and then aggregated to an IRUA territory level by means of averaging ...”
♣ Paragraph #3: “Based on the TNM Classification of malignant tumors, lung cancer cases were classified as advanced stage (TNM III and IV) and early stage (TNM I and II) ...”
♣ Paragraph #4: “The deaths that occurred in Brazil between 2011 and 2015 due to lung câncer (ICD-10, C33-34) [16] were collected from the Mortality Information System (MIS) ...”
♣ Paragraph #5: “For the correction of the number of deaths, the methodology proposed by Santos and Souza [18] was followed ...”
♣ Paragraph #6: “The crude and adjusted mortality rates (AMR) (per 100,000 inhabitants) were calculated for each IRUA ...”
♣ Paragraph #7: “The explanatory variables were classified into demographic and socioeconomic indicators ...”
♣ Paragraph #8: “Demographic and socioeconomic variables for 2010 were obtained from the Brazilian Atlas of Human Development (UNDP) ...” At the end of this last paragraph, keep the sentence already in lines 162-163: “Table 1 presents the study variables and corresponding descriptions ...”

The order of the paragraphs was modified as suggested.

⎫ Below the paragraphs of this Subsection, would then come Fig 1 (Flowchart) and Table 1 (Response and exploratory variables).

The figure and table were placed next to the text in which they are mentioned.

⎫ Line 118: describe the term “lung cancer” in italics.

The italic format was used to highlight the expression that was replaced by lung cancer in the text.

⎫ Lines 122 and 125: change the acronym “RHC”, which is in Portuguese, to “HCR” (Hospital Cancer Records).

The term has been changed to Integrator of Hospital-Based Cancer Registries (Integrator-HBCRs) as requested.

⎫ Line 129: replace the term “for 2011-2015” by “for each year”, as the way it is repeated in relation to line 127, where the years were already mentioned.

The term has been changed.

⎫ Line 130: replace the term “residence” by “residence address”.

The term has been changed.

⎫ Lines 131 and 133: the excerpt “For the correction of the number of deaths” was repeated. I believe it can be deleted from line 133.

The sentence has been excluded.

⎫ Line 135: does the word “state” mean “Federated Unit”? If so, it would be better to substitute by this last term.

The term has been replaced to Federated Unit (FU).

⎫ Lines 135 and 138: replace the acronym “SIM” by “MIS” (as defined this acronym in line 128).

The term has been changed.

⎫ Line 146: the acronym UNPD refers to the Organ: “United Nations Development Programme”, which needs to be properly described in full.

The acronym has been corrected.

⎫ Lines 147 and 148: the abbreviations “CNES” and “ANS” are in Portuguese; check the possibility of translating them into English, as was done for most acronyms in the text.

The authors chose to keep the abbreviations in Portuguese as they are Brazilian data sources. Access to these sources by readers may be impaired if we translate the abbreviation.

⎫ Line 154: replace “case selection” by “cases selection”.

The term has been changed.

⎫ Line 156 (title of Fig 1): change the acronym “RHC”, which is in Portuguese, to “HCR” (Hospital Cancer Records).

The acronym has been changed to HBCRs.

⎫ Table 1:

♣ Replace “IRHC” with “IHCR” (Source of the second response variable).

The term has been changed to Integrator-HBCRs.

♣ Description of first variable: replace “and standard world population” by “based on standard world population”.

The text has been changed as requested.

♣ Replace “Explanatory (Contextual)” by “Explanatory Variables (Contextual)”.

The term has been changed.

♣ In the descriptions of the explanatory variables, for some variables it is described that the population is from the year 2013, and for others it is not. Standardize.

The table was standardized and the information about the year of the population used in the calculation of all indicators remains in the eighth paragraph of the item "Study variables and data sources".

♣ In the description of the variable “Density of family health teams” the term “multiplied by” appears twice.

The description has been corrected.

♣ Consider revising the acronyms “CNES” and “ANS”, if they are changed in the rest of the text.

The authors chose to keep the abbreviations in Portuguese as they are Brazilian data sources. Access to these sources by readers may be impaired if we translate the abbreviation.

⎫ Line 176: replace “auto-correlation” with “autocorrelation” (no hyphen).

The term has been changed.

⎫ Lines 187-188: rewrite the sentence: “The validation of Moran’s I was carried out by a random permutation test, with 99 permutations” by: “The statistical significance of Moran's I was verified by a random permutation test, with 99 permutations”.

The sentence has been rewritten.

⎫ Lines 199-201: replace the phrase: “The multivariate analysis of lung cancer mortality included the explanatory variables that showed a statistically significant correlation with the response variable and non-colinear variables (correlation<0.7).” by: “The multivariate analysis of lung cancer mortality included explanatory variables that showed a statistically significant correlation with the response variable and that were not collinear with each other (correlation < 0.7).”

The phrase has been changed to:

The multivariable analysis of lung cancer mortality included explanatory variables that showed a statistically significant association with the response variable and that were not collinear with each other (correlation < 0.7).

⎫ Line 201: replace “log” with “lag”. The model is called “spatial lag” (not “log”).

The text has been corrected.

⎫ Lines 199-205: the authors explain how the multivariate analyzes were performed. In the first review, I had asked “why was multiple analysis done only for mortality, but not for the proportion of lung cancer cases with advanced diagnosis?” - and the authors replied: “The moderate spatial dependence of the proportion of diagnoses in advanced stage of lung cancer (Moran’s I = 0.37) and the weak correlations with the explanatory variables studied (as seen in figures 3 and 4) made it impossible to design a multiple analysis model.” I consider this information relevant to include in the text, perhaps in the limitations item, at the end of the discussion.

The information has been included in the limitations item.

5. Results:

⎫ Lines 215 and 217: standardize two decimal places in the 95% CI values.

The CI has been standardized as requested.

⎫ Line 227: replace “19.3%” with “19.36%” (two decimal places).

The information has been replaced to 19.36%”.

⎫ Lines 230-231: change sentence: “For the combined AMR for men and women, the average was 12.82 (SD 5.12) deaths per 100,000 inhabitants.” by: “The combined AMR for men and women was 12.82 (SD 5.12) deaths per 100,000 population.” (I believe the authors did not average the rates for men and women, which would be wrong, but instead calculated the rate for the entire population of men and women evaluated).

The sentence has been changed as required.

⎫ Line 231: where a new sentence starts - “The Itaberaba and Bom Jesus da Lapa ...” – break into another paragraph.

The text has been modified as suggested.

⎫ Line 236: “A spatial autocorrelation was observed (Moran’s I 0.50, p=0.01) with the formation ...”– does this autocorrelation refer to AMR? If so, consider rewriting: “AMR presented significative spatial autocorrelation (Moran’s I 0.50, p=0.01); with the formation ...”

The text has been rewritten as requested.

⎫ Line 255: replace “Figure 4” with “Fig 4”, to keep the same pattern as the titles of the other figures.

The term has been changed.

⎫ Line 259: replace “correlation” with “spatial correlation”.

The term has been changed.

⎫ Lines 260-262: “All correlations are statistically significant, except for correlation with “density of family health teams” (Moran’s I -0.02 p=0.28)” - consider switching to: “All correlations were statistically significant, except for the variable “density of family health teams” (Moran’s I -0.02 p = 0.28)”.

The text has been changed to: “All associations were statistically significant, except for the variable…”

⎫ Line 273: change “66%” to “66.3%” and reference Table 2.

The text has been changed.

⎫ Line 275: the variable name – “density of facilities licensed in oncology” is different from the nomenclature of the same variable in Table 2: “Density of Licensed Oncology Health Facilities”. Standardize this nomenclature throughout the text.

The term has been standardized to “density of facilities licensed in oncology”.

⎫ Lines 287-289: review the sentence: “The variable Density of Licensed Oncology Health Facilities showed a positive correlation with the studied outcome, although without statistical significance.” to: “The variable Density of Licensed Oncology Health Facilities showed no significant correlation with lung cancer mortality rate”.

The sentence has been changed to:

The variable Density of Facilities Licensed in Oncology showed no significant associated with age-adjusted lung cancer mortality rates.

6. Discussion:

⎫ Line 349: “In Forrest et al. [6]...” – change to: “In Forrest et al. study [6]..."

The text has been changed.

⎫ Line 351: exchange “inequalities social...” – by “social inequalities...”

The term has been modified as requested.

⎫ Line 405: instead of “mainly in territories of Rio Grande do Sul”, change to “mainly in the Rio Grande do Sul state”.

The text has been changed to “mainly in the Rio Grande do Sul FU…”

⎫ In the first review, I presented some considerations about some divergent results, related to the coverage of health plans and oncology services, comparing the results obtained through the different analysis techniques used. The authors responded brilliantly in the document to the reviewer; therefore, I strongly recommend that such considerations be added, albeit briefly, to the text of the article's discussion. Are they:
♣ Bivariate analysis showed a positive correlation between indicators of health insurance coverage and density of facilities licensed in oncology and the response variable adjusted mortality rates for lung cancer). But it was possible to see, through the maps, that there are clusters of high mortality in regions with low coverage of health plans, especially in IRUAs in the South region.
♣ In the regression model, the density of facilities licensed in oncology lost significance and the coverage of health plans showed an inverse correlation with lung cancer mortality. This discrepancy in results may be related to the levels of socioeconomic inequalities that exist even in the most developed regions, as discussed in the text. This makes access to public or private health services unequal among populations.
♣ Changes in address, in search of treatment, can also move part of the population with higher income to other regions with other health services, including private ones. Likewise, people who need access to health services through the SUS can migrate to places with a greater offer of public services. Both situations can contribute to the reduction of health plan coverage in regions with high mortality rates.

The considerations were added.

⎫ Lines 424-432: regarding this paragraph on the quality of health services, which cites the study by Kaliks et al., I suggest that the authors rewrite the paragraph incorporating their explanation in the document for reviewers, as follows:
♣ The study was carried out with 52 cancer centers linked to the Unified Health System (SUS). The data referring to the treatment were compared to what is recommended by the Ministry of Health, through the Diagnosis and Treatment Guidelines, and to the standard practiced in supplementary health, based on the Procedures List of the National Health Agency (ANS).

The text has been changed to:

The study by Kaliks et al [51] identified differences in the quality of treatment for the four most common cancers in Brazil, among the Public Health System (SUS), the assistance provided by health plan and the Therapeutic Guidelines established by the Ministry of Health. Of the 52 SUS cancer treatment centers investigated, only 29 followed the directives regarding lung cancer treatment. Eight presented higher standards, five were compatible, and 16 presented lower standards than suggested by the Therapeutic Directives. Furthermore, 19 SUS cancer centers presented standards below the recommended and practiced by health plans.

Line 431: specify what the abbreviation “SS” means in full.

The correct term is health plan.

Line 459: replace the term “can help” by “could help”.

The term has been changed.

Line 460: replace the term “could have” by “may have”.

The term has been changed.

7.Conclusions:
Lines 467-469: rewrite the sentence: “The proportion of advanced-stage diagnosis was weakly spatially correlated to socioeconomic and demographics indicators only.” by: “The proportion of advanced-stage diagnoses was weakly spatially correlated only with socioeconomic and demographic indicators.”

The text has been changed to:

A high proportion of advanced-stage lung cancer diagnoses was verified for almost the entire Brazilian territory for the study period. The proportion of advanced-stage diagnoses was weakly spatially associated only with socioeconomic and demographic indicators. However, age-adjusted mortality rates for lung cancer presented irregular distribution in the Brazilian IRUA, associated with territories that present higher incomes and lower coverage of health plans, regardless the supply of health facilities licensed in oncology.

Line 471: change the term: “independent of the offer” by “regardless the offer”.

The term has been modified as suggested.
